# Graphene-Based Sensor for Detection of Bacterial Pathogens

**DOI:** 10.3390/s21238085

**Published:** 2021-12-03

**Authors:** Santosh Pandit, Mengyue Li, Yanyan Chen, Shadi Rahimi, Vrss Mokkapati, Alessandra Merlo, August Yurgens, Ivan Mijakovic

**Affiliations:** 1 Department of Biology and Biological Engineering, Chalmers University of Technology, 412 96 Göteborg, Sweden; pandit@chalmers.se (S.P.); yanyanc@chalmers.se (Y.C.); shadir@chalmers.se (S.R.); mokkapativrss@gmail.com (V.M.); alessandra.merlo21@gmail.com (A.M.); 2 Department of Microtechnology and Nanoscience, Chalmers University of Technology, 412 96 Göteborg, Sweden; mengyue.lee@gmail.com (M.L.); yurgens@chalmers.se (A.Y.); 3 Novo Nordisk Foundation, Center for Biosustainability, Technical University of Denmark, 2800 Kongens Lyngby, Denmark

**Keywords:** graphene, sensors, *Pseudomonas aeruginosa*, *Staphylococcus epidermidis*, biofilms

## Abstract

Microbial colonization to biomedical surfaces and biofilm formation is one of the key challenges in the medical field. Recalcitrant biofilms on such surfaces cause serious infections which are difficult to treat using antimicrobial agents, due to their complex structure. Early detection of microbial colonization and monitoring of biofilm growth could turn the tide by providing timely guidance for treatment or replacement of biomedical devices. Hence, there is a need for sensors, which could generate rapid signals upon bacterial colonization. In this study, we developed a simple prototype sensor based on pristine, non-functionalized graphene. The detection principle is a change in electrical resistance of graphene upon exposure to bacterial cells. Without functionalization with specific receptors, such sensors cannot be expected to be selective to certain bacteria. However, we demonstrated that two different bacterial species can be detected and differentiated by our sensor due to their different growth dynamics, adherence pattern, density of adhered bacteria and microcolonies formation. These distinct behaviors of tested bacteria depicted distinguishable pattern of resistance change, resistance versus gate voltage plot and hysteresis effect. This sensor is simple to fabricate, can easily be miniaturized, and can be effective in cases when precise identification of species is not needed.

## 1. Introduction

Infectious diseases caused by pathogenic bacteria are one of the serious public health concerns and have a significant socioeconomic impact [1]. Bacterial infections such as nosocomial infections, tuberculosis, diarrhea, pneumonia, etc. generate a major burden to healthcare systems and affect millions of patents globally each year [2,3]. The early and reliable diagnosis of these infections is one of the key measures for correct treatment of such diseases and reducing patient suffering. The failure in treatment of the bacterial infections can be detrimental and can even lead to sepsis and death. Currently, the widely used diagnosis technique are polymerase chain reaction (PCR)-based methods, DNA microarrays, DNA sequencing technology, ELISA, staining, isolation, cell culture, and biochemical tests [4,5,6,7,8]. Most of these methods are quite complex, time consuming, involve multiple steps and require costly and high-precision instruments that rely on cumbersome procedures. They also suffer from false-positive results due to cross reactivity. Recently, several new fluorescent- and electrochemical sensors have been developed for rapid detection of pathogens [9,10,11]. However, the alternative methods also require additional chemical mediators such as fluorophores or redox agents for signal generation. Hence, there is a need for diagnostic devices which are easy to operate, compatible with clinical laboratories, and able to produce reliable results rapidly.

The interest towards the development of nanomaterials-based biosensors is rapidly emerging for sensitive and selective detection of pathogenic bacteria and viruses [12,13,14,15]. Among diverse nanomaterials, graphene is gaining attention in biosensor development, due to its remarkable properties [16,17,18]. Graphene is a two-dimensional sheet of sp^2^-bonded carbon atoms arranged into a honeycomb lattice. Graphene has a large surface area (2630 m^2^/g), which is beneficial for mediation of interaction with biomolecules [17]. The π orbitals of graphene provides a basis for sensitivity to the biomolecules capable of π–π interactions [19]. Furthermore, unique changes in the density of state (DOS) of graphene was observed due to the π–π interaction with aromatic molecules suggesting the potential of graphene not only for the detection of adsorption or desorption of molecule but also to identify the type of molecular complexes formed on graphene [20]. Hence, graphene-based sensors can be ideal for the detection of bacterial cells, which possess a negative surface charge. Indeed, graphene field-effect transistors (FET) have been successfully used for detection of proteins and DNA molecules [21]. The electrochemical biosensors based on impedimetric technology have attracted significant attention. These sensors rely on detection of changes in impedance of the sensor and have several critical advantages: rapid detection, high sensitivity, cost effectiveness, and label free detection [22]. Generally, the growth or adhesion of bacteria on the surface of the sensor are expected to alter its overall conductivity [23]. Such simple electrochemical sensors are not capable of differentiating among thousands of known bacterial species. Nevertheless, the selectivity of these sensors can be optimized by functionalizing with antibodies, bacteriophages, and synthetic materials, which are selective to specific bacterial cells [24,25]. Most of the published reports on FET sensors have successfully demonstrated some selectivity in detecting and quantifying different bacterial cells in solutions [26,27,28,29]. Especially, functionalized graphene sensors have been proven to be very selective to bacterial cells and able to detect the cells at very low concentrations. By contrast, pristine graphene without functionalization could also be used for sensitive detection of bacterial biofilm development in cases when selectivity is not an issue. However, there are no reports showing detection and differentiation of bacterial cells using non-functionalized graphene-based sensors.

Bacterial cells typically range in size from 0.5 to 5 µm, with distinct morphology depending on the group of bacteria [22]. The shapes of bacterial cells include spherical cocci such as *Staphylococcus epidermidis*, rod-shaped cells bacilli such as *Escherichia coli*, and spiral shape of *Spirillum volutans*. Most of the bacterial cells are encapsulated by a cell wall composed of peptidoglycan made of negatively charged N-acetylglucosamine and N-acetylmuramic acid. On the surface of bacteria, one can also find lipids, surface proteins, and glycoproteins [30]. The group of Gram-positive bacteria contains a thicker layer of peptidoglycans, whereas Gram-negative bacterial cells contain a thinner layer of peptidoglycan covered in an outer membrane and decorated with lipopolysaccharides and proteins [30,31]. Furthermore, some bacterial cells have tail-like structures known as flagella, which are used for generating motile force. Different bacterial cells have different adhesion ability to surfaces, which depends on their growth time, motility and surrounding hydrodynamic conditions [32,33]. Furthermore, bacteria sometimes produce different types of exopolymers, toxins, and other chemicals upon surface attachment [32,33]. These chemicals can affect the conductivity of graphene in different ways thereby giving some degree of specificity in detection. Considering this possibility, we asked whether a simple FET sensor based on pristine graphene could detect and distinguish between different types of bacterial cells. We demonstrated that several bacterial species can be detected and to some extent differentiated by our sensor, due to their different growth characteristics. We concluded that this type of sensor can be useful in cases when precise identification of species in a complex mixture is not needed.

## 2. Materials and Methods

### 2.1. Chip Fabrication

The method for sensor chip fabrication is demonstrated in Figure S1. On the SiO_2_/Si substrate, a layer of parylene was deposited through sublimation and pyrolysis of parylene-N dimer, for a final thickness of 150 nm of the polymer. The Si substrate was used as a back-gate electrode to electrostatically change the overall charge concentration in graphene assuming the parallel-plate-capacitance configuration, with the combination of SiO_2_ and parylene as the gate insulator. Small openings in the parylene layer were formed by photolithography and etching in oxygen plasma, followed by deposition of gold-on-chromium thin films, for the gold electrodes to firmly adhere to the primary substrate. Briefly, a layer of positive photoresist S1813 was spin coated at 3000 rpm and soft baked at 95 °C for 2 min. The pattern was generated by UV-light exposure through a photo mask and revealed by immersing the chip in Microposit™ MF319 developer for about a minute. The parylene was etched away by the O_2_ plasma at 40 watts for 3 min, in the unprotected by the photoresist pattern area. The photoresist was then removed by acetone and isopropyl alcohol (IPA), and blow-dried by nitrogen gas. The electrodes to graphene were fabricated by a double-layer lift-off photolithography. Briefly, a ~320-nm thick layer of LOR3A was spin-coated on the substrate at 3000 rpm, followed by soft-baking at 130 °C for 10 min. A second layer of the positive photoresist S1813 was spin coated at 3000 rpm, followed by soft baking at 95 °C for 2 min. The gold thin film (180 nm) was achieved by evaporation and to improve the adhesion of gold to the substrate, a thin layer of chromium was used (5 nm). The lift-off process was carried out first in acetone, then in MF319, to remove LOR3A, and finished by rinsing the chip in DI-water.

Graphene was grown on a 25-µm thick copper foil by CVD. Two layers of poly (methyl methacrylate) (PMMA) were spin-coated on the foil at 1000 rpm and dried at 50 °C for 30 min. A frame support cut out from a thermal-release plastic foil was attached to the copper foil to ease handling of fragile graphene membrane later on [34]. The copper foil was etched away by ammonium persulfate (APS). Graphene-on-PMMA “sandwich” was then placed onto the chip pre-covered by a droplet of IPA. After IPA was dried off, the PMMA was removed from the graphene by dipping the chip into acetone for 15 min at room temperature + another 15 min in a fresh acetone at 50 °C, followed by the final cleaning in IPA and blow-drying. After this step, graphene covered the whole chip area including the metal electrodes. Another photolithography was used to pattern graphene into a small bar-shape structure with several contacts reaching to the metal leads. Graphene was pre-coated (protected) by PMMA (2A-PMMA, spin coating at 6000 rpm, soft-baking at 160 °C for 3 min, all resulting in a ~60-nm-thick layer), to avoid parasitic doping from S1813, which results in worsen characteristics of graphene. To be able to use acetone for cleaning the structures from PMMA, an intermittent layer of acetone-insoluble LOR3A was spin-coated on top of the PMMA layer at 4000 rpm and pre-baked at 130 °C for 10 min. Finally, S1813 was spin coated at 4000 rpm, then pre-baked at 95 °C for 2 min, giving the final thickness of 1.3 µm. After UV-light exposure and photoresist development, the unprotected by the S1813/LOR3A pattern PMMA layer and graphene were etched in O_2_ plasma at 100 W for 1 min. Then the substrate was flood exposed by UV light for 40 s, so that both the LOR3A and the remaining photoresist can be dissolved in the developer (~30 s). To isolate the metal electrodes from contacting bio-active mixtures, which might affect the bacterial growth, a protection layer covering everything, but the graphene structure was created by patterning a layer of LOR3A (300 nm) soft-baked at 130 °C for 10 min, using another layer of S1813. Then, the PMMA thin film on top of graphene was removed by acetone.

The developed devices were tested for their functionality and quality. The good-working devices were than mounted on a larger printed-circuit-board (PCB) support, which was shaped to mimic the usual for domestic electronics micro-SD card layout. The gold electrodes of the sensor were connected to the copper lines of the PCB by using thin copper wires and conducting silver paint. The wires, the gold pads and some of the PCB lines were isolated from the environment by a bio-compatible PDMS (poly-dimethyl siloxane) layer. All-in-all, only graphene parts of the devices were in contact with bio-active mixtures, which minimized the number of stray parameters to consider. This sensor layout also allowed for their quick replacement in the home-made setup with multichannel read-out electronics. The electronics was designed for simultaneous measurements of the four-probe resistances of two graphene sensors on a single chip as functions of time and the back-gate voltage.

### 2.2. Culture Media and Bacterial Growth

The pathogenic bacteria, *Pseudomonas aeruginosa* PA01 (*P. aeruginosa*) and *Staphylococcus epidermidis* ATCC 35984 (*S. epidermidis*) were used in this study. Tryptic soy broth (TSB)/agar was used to grow *S. epidermidis* and lysogeny broth (LB)/agar was used to grow *P. aeruginosa*. The single colony of each bacterial strain was inoculated to respective media broth and incubated overnight on shaking incubator at 37 °C (300 rpm) to prepare inoculum. Gram positive bacteria (*S. epidermidis*) and gram-negative bacteria (*P. aeruginosa*) were used in the experiments. For that, 100 µL from overnight grown bacterial inoculum was sub-inoculated to 5 mL of fresh medium and incubated for 9 h in shaking incubator at 37 °C. To monitor the growth, the optical density of the bacterial culture was measured (600 nm) in the interval of 1 h.

### 2.3. Set-Up and Evaluation of Bacterial Sensing Ability

Once the proper functioning of the biosensor was adequately tested by measuring of transfer curves (the resistance *R* as a function of the back-gate voltage *V*g), the device was further used for detecting bacteria. First, a six-well plate was placed inside an incubator with a constant temperature maintained at 37 °C, to allow bacterial cells to grow. The cover of the well-plate was substituted with a layer of aluminum foil covering the plate to minimize evaporation of the liquid medium and any possible contamination. A small window in the aluminum foil allowed the dipping of the biosensor in the growth medium containing the bacterial inoculum. The device was than connected to the read-out electronics and the value of resistance was measured every 30 s for the entire duration of the experiment. The back gate voltage (Vg) was kept off during the measurement. For the first five minutes of the recorded measurements, the biosensor was kept outside the bacterial solution, at room temperature, to have a reference value of the initial resistance of the device prior to the dipping into the solution. The device was then carefully dipped into 12 mL of the growth medium containing bacterial inoculum through the small window in the aluminum foil, making sure the exposed graphene area is entirely submerged. Following the dipping, sensor chip was kept in the bacterial solution for 5 h, considered to be an adequate time for the bacteria to grow and form a biofilm at the graphene surface. After five hours, 200 μg/mL of ciprofloxacin was added to the solution, without the extraction or any movement of the biosensor, to kill the bacteria and stop their replication. The quantity of added antibiotic was appropriately chosen to guarantee the complete deactivation of bacteria. The measurement was continued for three hours after the antibiotic addition. Similarly, changes in resistance value were also measured with the gate voltage on for *P. aeruginosa* and *S. epidermidis*. A scanning electron microscope was used to examine the adhesion of bacterial cells and biofilm formation on the sensor device after 5 h of bacterial growth. Briefly, adhered bacteria on the sensor chips were fixed with 3% of glutaraldehyde for 2 h and dehydrated with graded series of ethanol (40, 50, 60, 70, 80, 90, and 100%) for 10 min each. The samples were dried overnight at room temperature. The dried samples were sputter coated with gold (5 nm) and examined by using a JEOL JSM 6301F (Carl Zeiss AG, Jena, Germany).

## 3. Results

### 3.1. Characterization and Validation of the Sensor Chip

**Figure 1:**
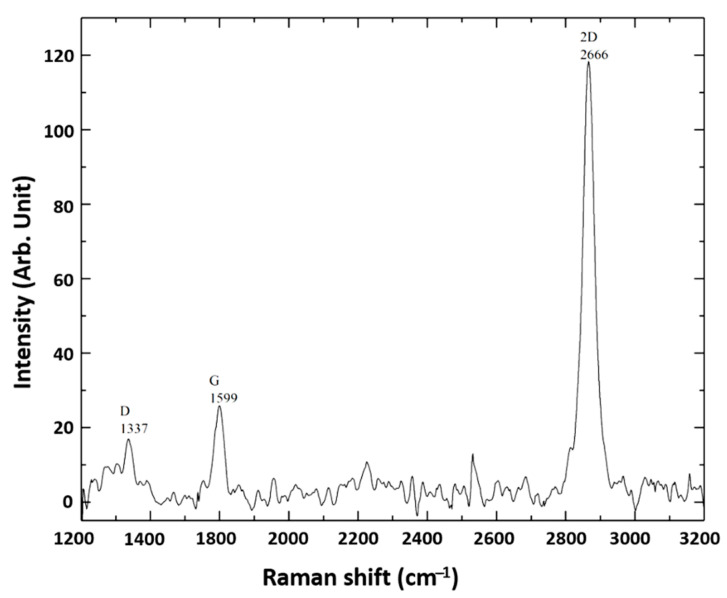
Raman spectrum of CVD-grown graphene on copper substrate.

**Figure 2:**
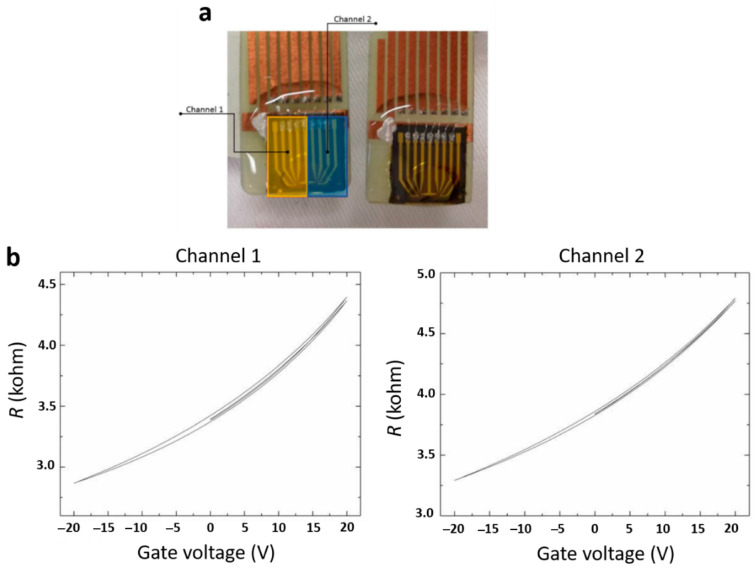
Representative (**a**) image of operational sensor chip and (**b**) resistance (R) vs. gate voltage (V) characteristics of the biosensor, for both channels of the device.

The quality of CVD-grown graphene on copper foil was characterized by the Raman spectroscopy. As shown in Figure 1, the two peaks characteristic for pristine graphene were visible, confirming the purity and monolayer form of graphene on the copper surface. The fabricated devices contained two sensing elements on the same substrate chip, named Channel 1 and Channel 2 (Figure 2a). This allowed for duplicate measurement at the same conditions, enabling statistical validation of the results and timely detection of the measurement errors. R-VG characteristics were examined to verify the proper functioning of the two channels of the biosensor (see Figure 2b). R increased with VG in the whole range VG < 10 V [35], indicating the residual *p*-doping of graphene most likely due to some chemical residues adsorbed at the surface of graphene or proximity to electronegative molecules nearby (like OH-groups that hydrophilic SiO_2_ is normally covered with). Figure 2b also underlines the presence of a slight hysteresis during VG-sweep, illustrating the charging and discharging process, possibly involving a slow charge transfer to and from molecules in the vicinity of graphene.

### 3.2. Bacterial Growth Pattern

**Figure 3:**
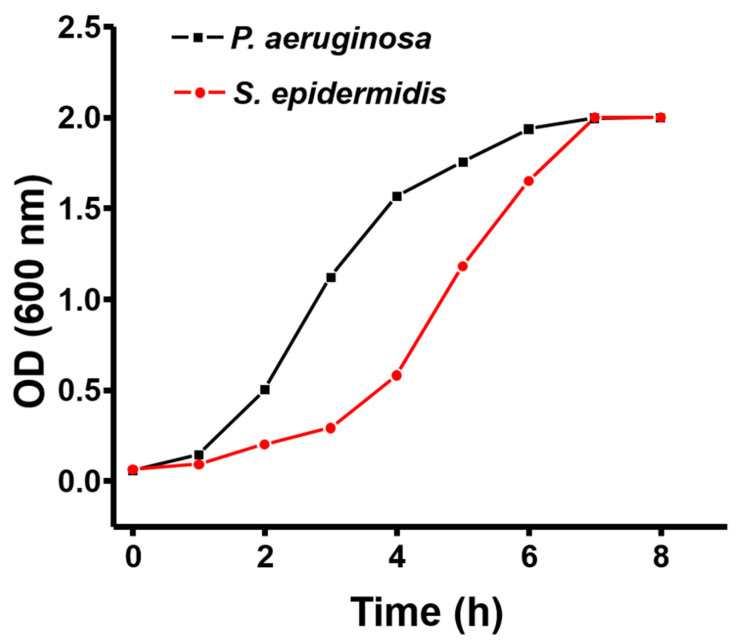
The growth pattern of *P. aeruginosa* and *S. epidermidis*. *P. aeruginosa* was grown in a sterile tube containing LB broth and *S. epidermidis* grown in a sterile tube containing TSB broth. The optical density of culture was measured for 8 h with the interval of 1 h.

**Figure 4:**
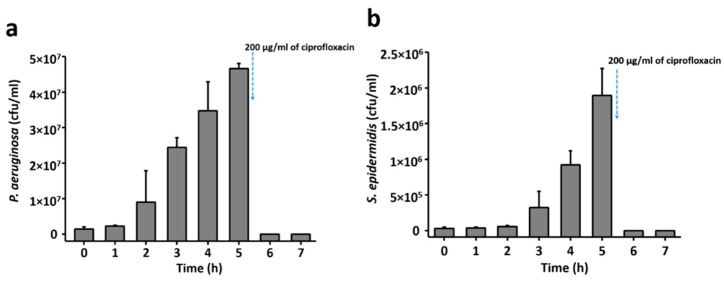
Number of (**a**) *P. aeruginosa* and (**b**) *S. epidermidis* cells with respect to growth time in liquid culture. After 5 h of growth, 200 µg/mL of ciprofloxacin was added to the culture medium.

Prior to exposing the sensor to bacteria, the growth profile of bacteria in the liquid culture was examined by measuring the optical density (OD) over time. Figure 3 shows the growth profile of *P. aeruginosa* and *S. epidermidis* in their respective culture medium, over the period corresponding to the sensing experiment. Both bacterial strains were observed to follow a normal growth pattern in our experimental setup. For both of the tested bacterial strains, the first 2 h of culture corresponded to a lag phase, where bacteria adapt to a new environment and the growth is not significant. After this, bacterial cultures entered the log phase of rapid growth, where *P. aeruginosa* was observed to grow faster than *S. epidermidis*. The log phase lasted from 3 to 5 h for *P. aeruginosa* and 3 to 6 h for *S. epidermidis*. The OD measurements correspond to only a semi-quantitative representation of bacterial growth. Determination of number of viable bacterial cells with respect to growth time is crucial to evaluate the sensitivity of sensors. Hence, to determine the number of bacterial cells, a fraction of bacterial suspension was plated to agar plates to count bacterial colony forming units (CFUs) (see Figure 4).

The number of *P. aeruginosa* and *S. epidermidis* cells with respect to growth time is presented in Figure 4a,b, respectively, in terms of colony forming unites. As shown in Figure 4, no significant increase in the number of bacterial cells was detected until 3 h of growth. This is compatible with the result obtained from the growth-pattern analysis (Figure 3) where a lag phase of bacterial growth was observed. Significant growth of bacterial cells was observed from 3 h of growth for both strains. The addition of ciprofloxacin after 5 h of growth reduced the number of viable bacteria to zero. 

### 3.3. Sensing of Different Bacterial Strains

**Figure 5:**
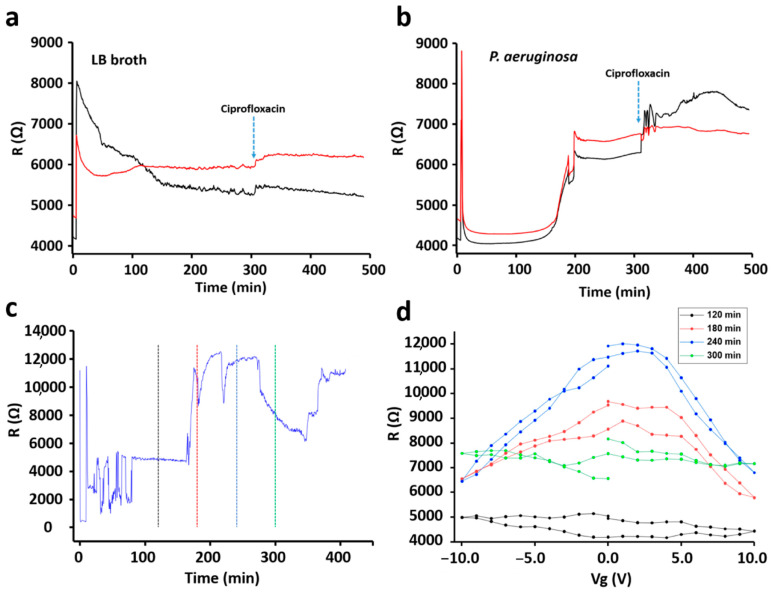
The resistance-versus-time plots for LB medium (**a**) and *P. aeruginosa* (**b**) without gate voltage (Vg). Resistance -versus-time plot for *P. aeruginosa* in the presence of Vg (**c**). Resistance-versus-Vg plot at different time points (**d**) indicated in (**c**) by the vertical dashed lines color matched with R(Vg)-curves in (**d**).

**Figure 6:**
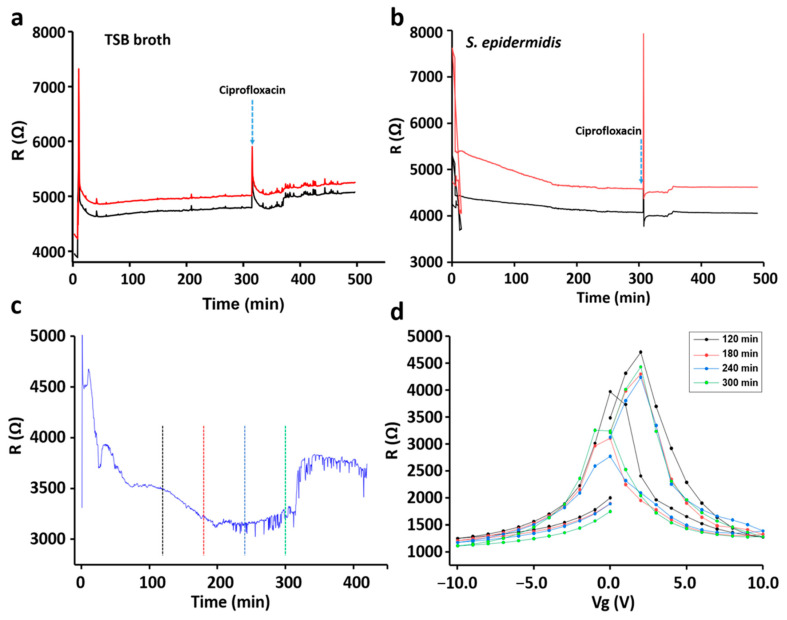
The resistance-versus-time plots for TSB medium (**a**) and *S. epidermidis* (**b**) without gate voltage (Vg). Resistance-versus-time plot for *S. epidermidis* in the presence of Vg (**c**). R(Vg) plots at different time points (**d**) indicated in (**c**) by the vertical dashed lines color matched with R(Vg)-curves in (**d**).

*P. aeruginosa* and *S. epidermidis* were used to evaluate the sensing ability of the developed sensor device. *P. aeruginosa* was used as a model for Gram-negative bacteria and *S. epidermidis* was used as a model for Gram-positive bacteria. The sensor chip was dipped into bacterial cultures growing in six well plates continuously for 8 h. Over the entire period, changes in electrical resistance of the graphene chip were measured with both conditions, with back gate voltage on and off. For all experiments, we first recorded the chip response in the appropriate growth medium without bacteria for 8 h, LB broth for *P. aeruginosa* (Figure 5a) and TBS broth for *S. epidermidis* (Figure 6a). All the experiments were performed at 37 °C. After the background-signal measurements, the same chips were washed and then used for sensing of bacteria. 

In all control reactions an increase in resistance can be seen soon after the exposure of the chip to the growth media (LB for *P. aeruginosa*). The signal then remains relatively stable for the next 5 h (Figure 5a). After 5 h, the antibiotic solution (200 µg/mL) was introduced to the medium, which generated a mild increase in chip resistance. After acquiring these background signals, sensor chips were exposed to a fresh media, containing the inocula of *P. aeruginosa* (Figure 5b). The detection procedure was the same as in the control experiments: 5 h at 37 °C to allow bacterial growth and thereafter the addition of an antibiotic. As shown in Figure 5b, the largest increase of the resistance was observed after 3 h of *P. aeruginosa* growth. This corresponded to the large increase in the number of bacterial cells, from about 9.0 × 10^6^ to 2.5 × 10^7^, in the period from 2 to 3 h of the growth (Figure 4a). The increment in the resistance pattern was observed to be similar when the resistance was measured with the back gate voltage on (Figure 5c, Figures S2a and S3a). A large increase of the resistance was observed around 3 h of *P. aeruginosa* growth even when the gate voltage was on. Later, the resistance versus the gate voltage, *R*(*V*g), was plotted by using the resistance-profile data obtained while *V*g was on during the measurement. As shown in Figure 5d, a small shift in the charge-neutrality point (CNP) towards the positive region was observed with *P. aeruginosa*. The consistency in the shift of CNP and small hysteresis caused by *P. aeruginosa* adhesion was confirmed by biological replicates (Figures S2b and S3b). Figure 6a shows changes in the resistance profile of sensor chip in the presence of TSB medium (used for *S. epidermidis*). Unlike *P. aeruginosa*, *S. epidermidis*, a Gram-positive bacterium, showed a different pattern. As shown in Figure 6b, a decreasing-resistance pattern was observed with respect to bacterial growth time. The decrease in resistance continued for 3.5 h and stayed in the stationary phase until the addition of an antibiotic. A similar pattern was observed with *S. epidermidis,* when the measurements were done with the back gate voltage on (Figure 6d and Figure S4a). As shown in Figure 6d, a small shift in CNP towards positive *V*g was observed and was similar with *P. aeruginosa*. However, a huge hysteresis was observed with *S. epidermidis* resistance-versus-gate voltage plot in comparison with *P. aureginosa*. Moreover, in other biological replicate, a slight shift of CNP towards negative *V*g was observed despite a similar decreasing-resistance pattern (Figure 6c and Figure S4a).

**Figure 7:**
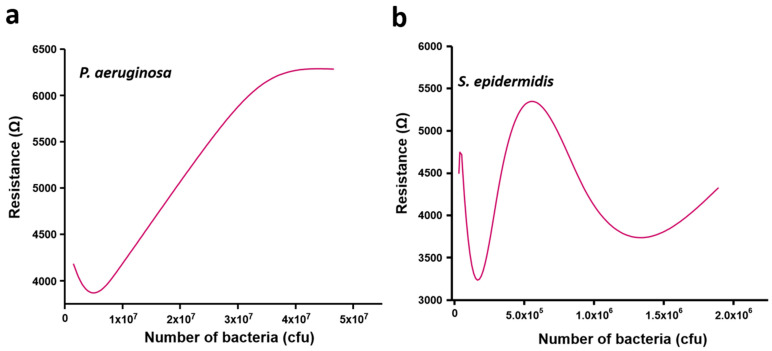
Resistance versus number of bacterial cells in terms of colony forming units (CFUs) for (**a**) *P. aeruginosa* and (**b**) *S. epidermidis*. The CFUs of bacterial cells were determined with the interval of 1 h until 5 h of cultivation (0–5 h). The obtained resistance value at 0, 1, 2, 3, 4, and 5 h were used to generate the plot of resistance versus CFU.

This again correlated with the slower growth of *S. epidermidis* compared to *P. aeruginosa* (Figure 3). The number of *S. epidermidis* cells increased from 6.0 × 10^4^ to 9.0 × 10^5^ between 2 and 4 h of growth (Figure 4b). As shown in Figure 7, the resistance versus CFU plot shows the increase of resistance with the number of CFUs. The increase in resistance with *P. aeruginosa* was linear in the range of ~0.5 × 10^6^ to 3 × 10^7^ CFUs, followed by a sharp saturation (Figure 7a). By contrast, the resistance-vs-CFU profile for *S. epidermidis* was more complex, with a local minimum at 2 × 10^5^ and a maximum between 6–7 × 10^5^ CFUs (Figure 7b). Some bacterial strains produce acids during their growth, which can influence the resistance pattern. The pH of the bacterial culture after 5 h of growth is presented in Table 1. For both tested bacterial strains, the pH after 5 h of bacterial growth was found to be similar to the initial pH. This suggests that the observed pattern is not due to the pH changes in the culture but is solely due to the adhesion of bacteria to the sensor surface, according to their distinct growth dynamics and changes in the environment.

**Figure 8:**
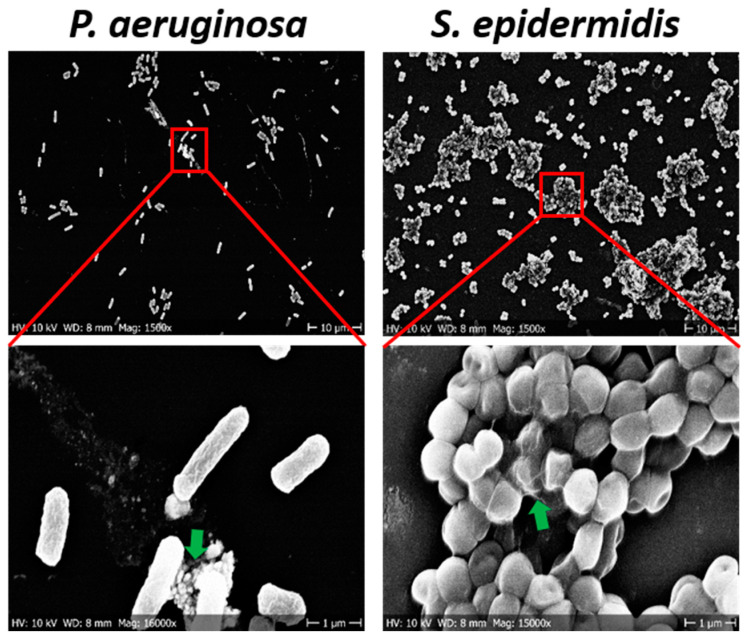
SEM images of *P. aeruginosa* and *S. epidermidis* biofilms after 5 h of growth on the sensor chip. The lower panel shows bacterial cells at a higher magnification. The green arrows depict the exopolymeric substances.

We further explored the chip surface with SEM, to assess the extent of bacterial adhesion followed by the biofilm formation after 5 h of bacterial growth. Figure 8 depicts the adhesion of bacterial cell at the sensor surface after 5 h of growth. Adhesion of bacterial cells at the surface in the form of exopolymeric substances was observed for all bacterial strains. However, for *P. aeruginosa* a large majority of cells were in single-cell stage, with very few multicellular clusters corresponding to early biofilms. By contrast, *S. epidermidis* cells were predominantly in large multicellular clusters. Hence, the unusual transitions in the resistance-vs-CFU profile for *S. epidermidis* can most likely be attributed to events triggering the later, and due to already more mature stages of biofilm formation, where correlation between the signal and CFU counts is completely lost due to loss of unicellularity.

## 4. Discussion

Biofilms are dynamic multicellular 3D structures developed by microorganisms [36,37]. The aggregation or adhesion of microbial cells on a biotic/abiotic surface is a key primary stage for such biofilm formation [38]. Such biofilms developed by pathogenic bacteria cause serious infections or sepsis. Hence, early detection of microbial colonization or biofilm formation on surfaces could provide an early diagnostic alarm, which would play a vital role in preventing serious infections. In this scenario, FET sensors are believed to have a significant potential as early diagnostic tools [39,40]. The sensor developed in this study was able to detect resistance changes upon microbial colonization and biofilm formation on its surface. The used bacterial strain *P. aeruginosa* is known to be electrochemically active with their own electron transfer mechanisms as like other gram-negative bacteria [41,42,43]. In addition to that the electrochemical activity of *P. aeruginosa* also related to production of phenazine derivatives [41,42,43]. However, there is no such information in electron transfer mechanism elucidated for *S. epidermidis* so far.

A clear trend in resistance change of graphene-based sensors with increasing number of bacterial cells in contact with the sensor surface has been reported before [44,45]. Functionalized graphene surfaces were mainly used in previous studies to detect some specific bacterial strains [22,39,44]. The efficiency of such sensors is mainly based on the conductivity changes of graphene sheets upon the interaction of antibodies attached to graphene and bacterial cells that bind to these antibodies. However, the direct interaction of bacterial cells with graphene surface also could change the electrostatic profile of graphene due to negative charge present in outer cell wall and membrane components, such as proteins and phospholipids. In addition to that, the adhesion of different bacterial cells with their distinct morphological features could result in different changes in the carrier density in graphene. The sensitivity of pristine graphene to such perturbations paves the way for very simple non-functionalized sensors.

In this study, we attempted to exploit this extreme sensitivity of pristine graphene to detect the formation of biofilms by different bacterial species. Our current sensing chip contained pristine graphene without any chemical mediators or functionalization with antibodies. Despite its simplicity, our sensing chip was able to detect formation of biofilms by *P. aeruginosa* and *S. epidermidis*. The changes in graphene resistance occurred only after the density of bacterial population reached to 10^5^–10^7^. This correlated very well with the known phenomenon of quorum sensing, a regulatory mechanism based on sensing population density, which triggers biofilm formation [46,47,48]. It should be noted that an optimal growth of bacterial cells is needed for surface colonization and to begin the biofilm formation. This was well reflected in our results that *P. aeruginosa* which grow relatively fast (Figure 3), could adhere to sensor chip earlier in comparison to *S. epidermidis* counterpart resulting in a rapid increase of resistance after about 3.5 h of growth (Figure 3 and Figure 5b). Hence, there can be a time difference in the primary adhesion and biofilm formation between various strains and this mostly depend on the growth profile, motility, and aggregation [38,49,50]. The surface colonization of *P. aeruginosa* is primarily mediated by their swimming or motile activity. Due to its motile behavior, *P. aeruginosa* can move even after the primary adhesion to surface, and this could be the reason for the small hysteresis observed in the *R*(*V*g) plot (Figure 5d, Figures S2b and S3b). Moreover, *P. aeruginosa,* being a rod-shaped bacterium, takes more area of graphene. When they move around, they create time-varying charge imbalance at the graphene surface, which could cause the hysteresis effect. Whereas for *S. epidermidis* as nonmotile bacteria it gets aggregated and adhered to the surface with the help of other biomatrix [32,51]. Furthermore, non-motile *S. epidermidis* get accumulated on the sensor surface and tend to form islands of microcolonies, supplying negative charge to the *p*-doped graphene thereby bringing the system closer to CNP, which should lead to the overall increase of resistance. However, the charge doping from the microcolonies is not spatially uniform in graphene resulting in a decrease of the maximum resistance at CNP. This compensating effect results in a slow and steady decrease in resistance with bacterial growth at zero *V*g (Figure 6b,c and Figure S4). In addition to that, high density of bacteria and accumulated micro-colonies (Figure 8) on the sensor surface may be responsible for the large hysteresis observed for *S. epidermidis*. Fluctuations in *R*(*V*g) accompanied by shifts in CNP were observed for independent biological replicates (Figure 6d and Figure S4b). The actual reason for such different behavior is not known. It might be due to the difference in density of adhered microcolonies to graphene surface for different biological replicates. Sometimes bacterial cells weakly attach to the surface, which may lead to detachment and reattachment of bacterial cells during the early stages of biofilm formation [52]. Hence, it is possible to obtain an irregular pattern of the resistance change during sensing of a biofilm formation in the continuous culture condition. This behavior can be seen more frequently for motile bacteria such as *P. aeruginosa*, which tend to attach and detach by themselves at the stage of early biofilm formation. Due to this fact, it is obvious to see such fluctuations in the *R*(*V*g) with each individual experimental setup. In this study, although small fluctuations in the *R*(*V*g) was observed with *P. aeruginosa*, the overall pattern of resistance increase with growth time and resistance-versus-Vg plots observed to be similar. It should also be noted that bacterial cells in biofilms release metabolic byproducts such as enzymes, proteins, ions, nucleic acids, and polymeric substances which could also alter the resistance with the time of biofilm growth. Difference in growth pattern and growth time with respect to different species could also be beneficial in a way that different bacteria, depending on the type of strain, availability of nutrients and culture condition, might need different incubation times to reach the detectable number of cells [49]. It was easier to distinguish between the motile Gram-negative (*P. aeruginosa*) and non-motile Gram-positive bacterium *S. epidermidis*, due to its enhanced propensity to form dens microcolonies in shorter time periods (Figure 5b and Figure 6b). It is not likely that such chips will be able to distinguish bacteria on a species level, even with very advanced signal-analysis approaches. However, they may be sufficiently sensitive and specific as early warning systems in diagnostics. In addition to that, administration of ciprofloxacin generated an interesting insight in the sensing mechanism of our chips. The applied concentration was clearly able to kill all bacterial cells (Figure 5). However, the chip resistance signal did not decrease after the addition of ciprofloxacin, which suggests that dead bacteria did not emit or change anything in terms of signal generation.

The growth patterns and growth time of the tested bacteria were different, and these differences were reflected in the pattern of resistance changes in our sensor response (Figure 3, Figure 5 and Figure 6). However, batch-to-batch differences in the quality of graphene as well as adhesion diversity of microbial cells to surface are observed to be the key challenges for reproducible and robust sensing. Despite being different in strain and morphology, most of the bacteria tend to provide sufficient charge that can shift CNP closer to zero *V*g.

As a future perspective, it would be interesting to test mixed bacterial cultures, to analyze the chip response. This would allow one to assess whether the chip response is a linear combination of the two curves of individual bacterial species, thus allowing the distinction of single species by de-convoluting the signal, or the signal from one bacterium would repress the other. Furthermore, it would be also meaningful to track the response of graphene chip by IR and Raman spectra upon the adhesion of different bacterial strains on this type of graphene sensor chip to understand the response, which could help to enhance the selectivity of the sensor. Hence, in future study we are planning to test the additional parameter such as IR and Raman spectra response of graphene after different stages of biofilm formation. In addition to that we will examine the changes in electric parameters while feeding the sensor with different frequencies of modulated signals. Furthermore, we are also developing the selective receptor of bacterial cells which can be attached to graphene to enhance the sensitivity of sensor for the detection of bacterial cells.

## 5. Conclusions

The obtained results in this study demonstrate the feasibility of using a label-free pristine graphene-based sensor to monitor early bacterial colonization and biofilm formation. The adhesion and biofilm formation by bacterial cells on the sensor surface were reflected by the change in the resistance pattern of the sensor. The detection limit of the developed sensor was around 10^5^ to 10^6^ CFU/mL and the sensing output was dependent on the different growth dynamics, adhesion to graphene, density of adhered bacteria, and microcolonies formation. Our sensor was not very sensitive to lower number of bacterial cells, but it showed a different signal output for *P. aeruginosa* and *S. epidermidis*. Further studies with more bacterial species should be performed to precisely define the selectivity of the sensor. Overall, we propose that a very simple sensor based on pristine graphene without any functionalization could potentially detect bacterial adhesion and biofilm development in cases when species specificity is not an issue.

## Figures and Tables

**Table 1:** pH before and after bacterial-culture growth.

Bacterial Strains	Culture pH (0 h)	Culture pH (5 h)
*P. aeruginosa*	6.99	7.06
*S. epidermidis*	7.18	7.09

## Data Availability

Not applicable.

## Supplementary Materials

The following are available online at https://www.mdpi.com/article/10.3390/s21238085/s1, Figure S1: Schematics of the sensor-chip fabrication. Figure S2: Biological replicate for resistance-versus-time plot for *P. aeruginosa* while Vg was on during the measurement. Figure S3: Biological replicate for resistance-versus-time plot for *P. aeruginosa* while Vg was on during the measurement. Figure S4: Biological replicate for resistance-versus-time plot for *S. epidermidis* while Vg was on during the measurement.
