# Supplementary material for: Graphene-Based Sensor for Detection of Bacterial Pathogens
Source: Sensors (Basel). 2021 Dec 3;21(23):8085. doi: 10.3390/s21238085 (PMC8662450; doi:10.3390/s21238085)
Supplement: Supplementary file 1 [file sensors-21-08085-s001.zip › sensors-1477014-supplementary.pdf]

sensors

MDPI

Article

Graphene-Based Sensor for Detection of Bacterial Pathogens

Santosh Pandit[1], Mengyue Li[2], Yanyan Chen[1], Shadi Rahimi[1], VRSS Mokkapati[1], Alessandra Merlo[1], August Yurgens[2], and Ivan Mijakovic[1,3]*

[1] Department of Biology and Biological Engineering, Chalmers University of Technology, 412 96 Göteborg, Sweden;
pandit@chalmers.se (S.P.); yanyanc@chalmers.se (Y.C.); shadir@chalmers.se (S.R.); mokkapativrss@gmail.com (V.M.); alessandra.merlo21@gmail.com (A.M.)
[2] Department of Microtechnology and Nanoscience, Chalmers University of Technology, 412 96 Göteborg, Sweden;
mengyue.lee@gmail.com (M.L.), yurgens@chalmers.se (A.Y.)
[3] Novo Nordisk Foundation, Center for Biosustainability, Technical University of Denmark, 2800 Kongens Lyngby, Denmark
* Correspondence: ivan.mijakovic@chalmers.se; Tel.: +46-(0)7-0982-8446

Citation: Pandit, S.; Li, M.; Chen, Y.; Rahimi, S.; Mokkapati, V.; Merlo, A.; Yurgens, A.; Mijakovic, I. Graphene-Based Sensor for Detection of Bacterial Pathogens. *Sensors* **2021**, *21*, 8085. https://doi.org/10.3390/s21238085

Academic Editor (s): Vojtěch Adam

Received: 08 November 2021
Accepted: 28 November 2021
Published: 3 December 2021

Publisher's Note: MDPI stays neutral with regard to jurisdictional claims in published maps and institutional affiliations.

Copyright: © 2021 by the authors. Submitted for possible open access publication under the terms and conditions of the Creative Commons Attribution (CC BY) license (http://creativecommons.org/licenses/by/4.0/).

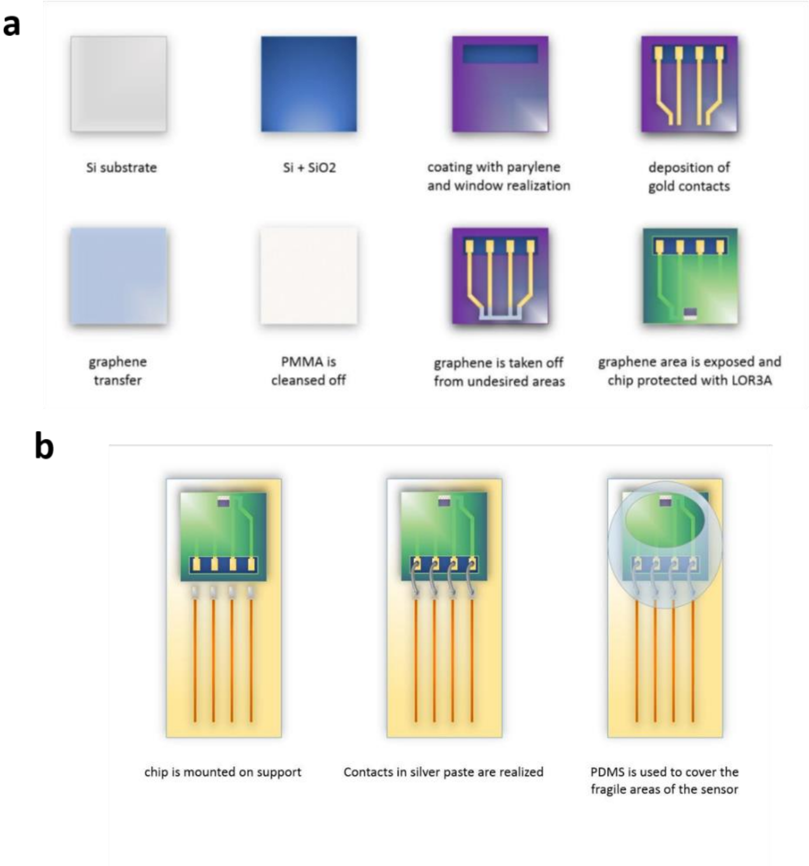

Figure S1. Schematics of the sensor-chip fabrication. a) The graphene transfer onto a chip with pre-patterned gold electrodes. b) Mounting of the sensor chip onto a larger support mimicking the micro-SD card layout, which allowed for a quick connection (and replacement) of the sensor into a corresponding socket of the signal-readout electronics.

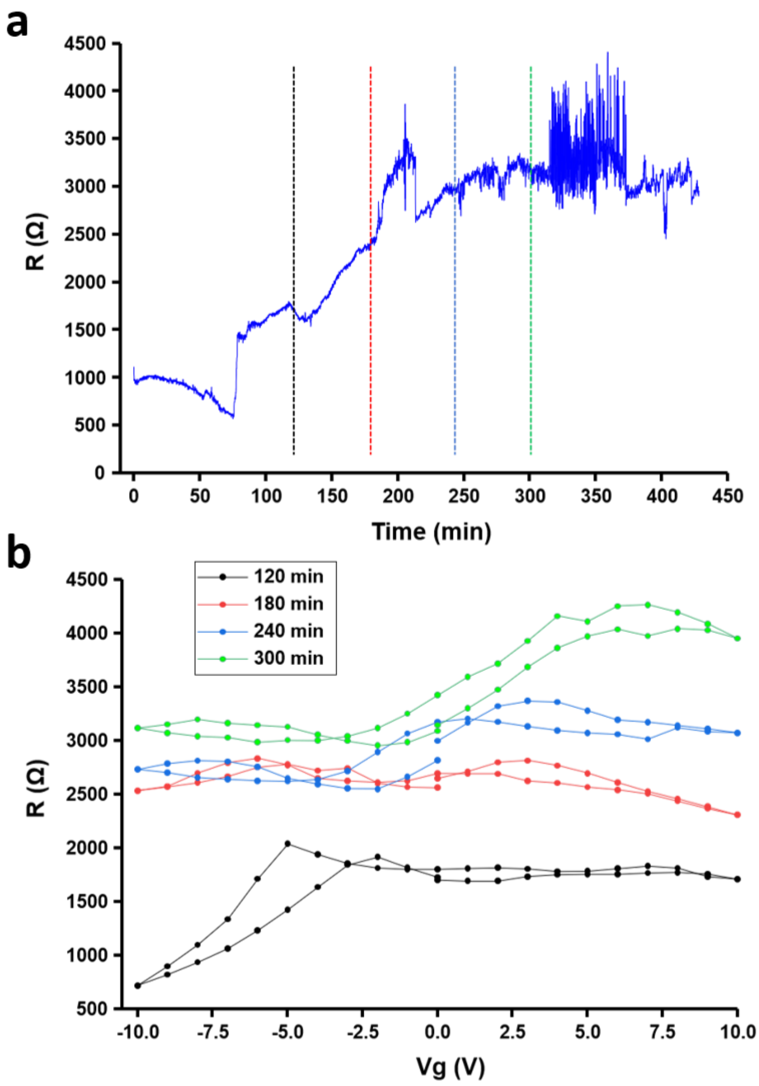

Figure S2. Biological replicate for resistance-versus-time plot for *P. aeruginosa* in the presence of Vg (a). R(Vg) plots at different time points (b) indicated in (a) by the vertical dashed lines color matched with R(Vg)-curves in (b).

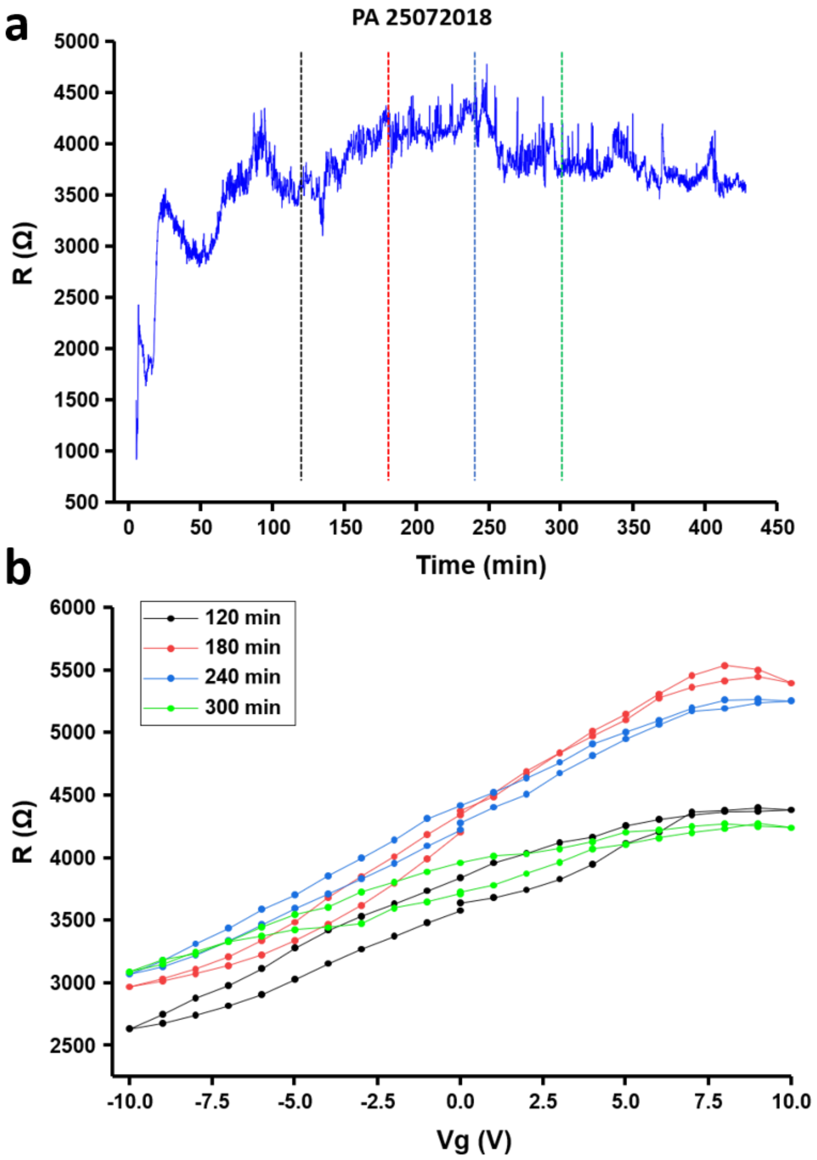

Figure S3. Biological replicate for resistance-versus-time plot for *P. aeruginosa* in the presence of Vg (a). R(Vg) plots at different time points (b) indicated in (a) by the vertical dashed lines color matched with R(Vg)-curves in (b).

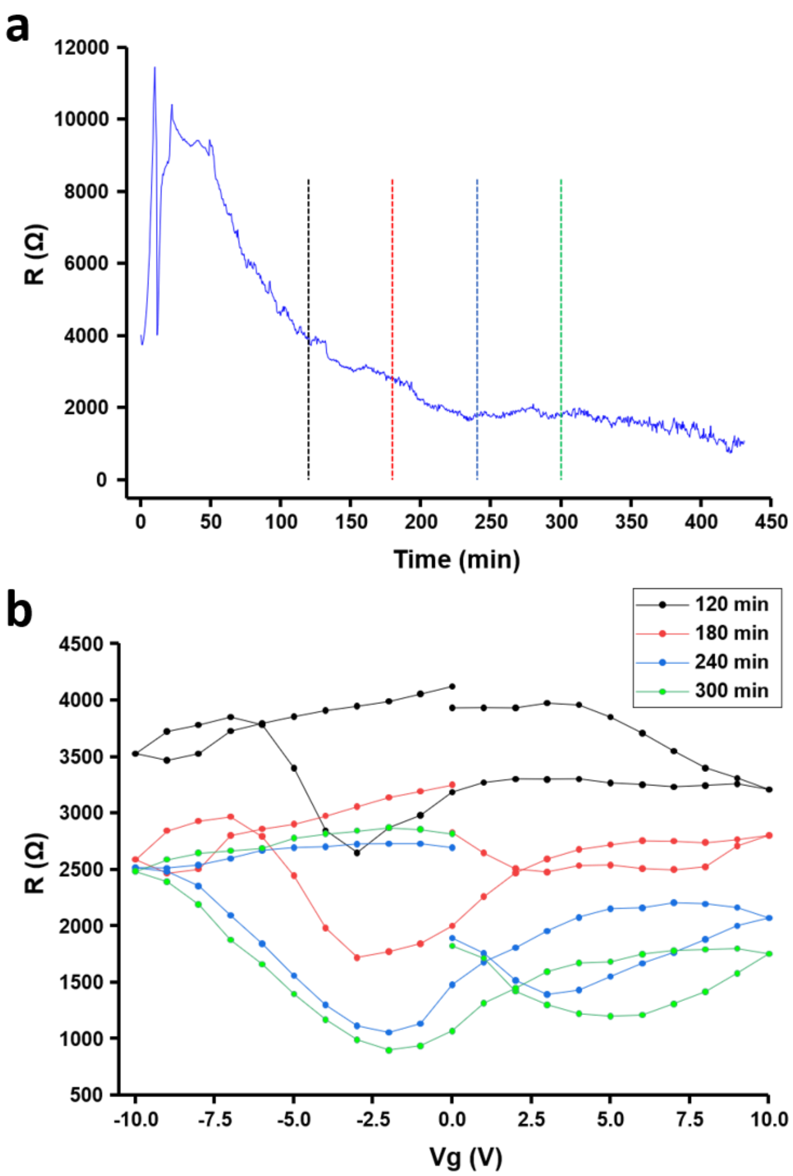

Figure S4. Biological replicate for resistance-versus-time plot for *S. epidermidis* in the presence of Vg (a). R(Vg) plots at different time points (b) indicated in (a) by the vertical dashed lines color matched with R(Vg)-curves in (b).
